# Progressive ossification due to retained surgical sponge after upper leg amputation: a case report

**DOI:** 10.4076/1757-1626-2-8592

**Published:** 2009-08-06

**Authors:** Irene C Kouwenberg, Jan Paul M Frölke

**Affiliations:** 1 Department of Surgery, Radboud University Medical Center, Nijmegen 690, The Netherlands

## Abstract

**Introduction:**

Numerous cases are described of patients in whom foreign objects were found after surgery. Foreign body granuloma caused by retained surgical sponge, also called gossypiboma, mostly occur in the abdominal cavity but very seldom in limbs.

**Case presentation:**

A 29-year-old Caucasian man presented with asymmetrical walking pattern and progressive pain in his leg, which was severely injured and amputated seven years before. A firm swelling of soft tissue with calcifications was localized in the stump. Roentgenogram and MRI showed a retained surgical sponge with calcifications. Open surgery was performed and a well-encapsulated, brownish soft-tissue tumour containing serous fluid was found in which the remnants of a surgical sponge of 40 × 40 centimeters was identified and removed. Infectious complications characterized the postoperative course for which multiple surgical procedures were needed to create a definitive healing of the stump.

**Conclusion:**

A surgical sponge left behind in an amputated leg may lead to fibroma, destruction, osteolysis and calcification. In our case the gauze lead to mild dysfunction of the prosthetic leg, asymmetrical walking pattern, phantom pain and calcification and osteolysis on roentgenogram.

## Introduction

Numerous cases are described of patients in whom retained surgical sponges were detected after surgery, months or even years after the initial operation. Retention of these foreign bodies may lead to formation of soft tissue mass with possible bony involvement [1]. Previous studies have suggested that a retained surgical sponge occurs in 1 per 1000 to 1500 abdominal operations [2]. Jordan et al. found the incidence of a retained sponge occurred in 1 per 5027 operations [3]. It is likely that these complications are underreported. A foreign body granuloma caused by a retained surgical sponge is called a gossypiboma [4,5]. Most gossypibomas are described in the abdominal cavity, with only very few cases of retained surgical sponges located in limbs [4]. It has never been described after amputation.

## Case presentation

**Figure 1:**
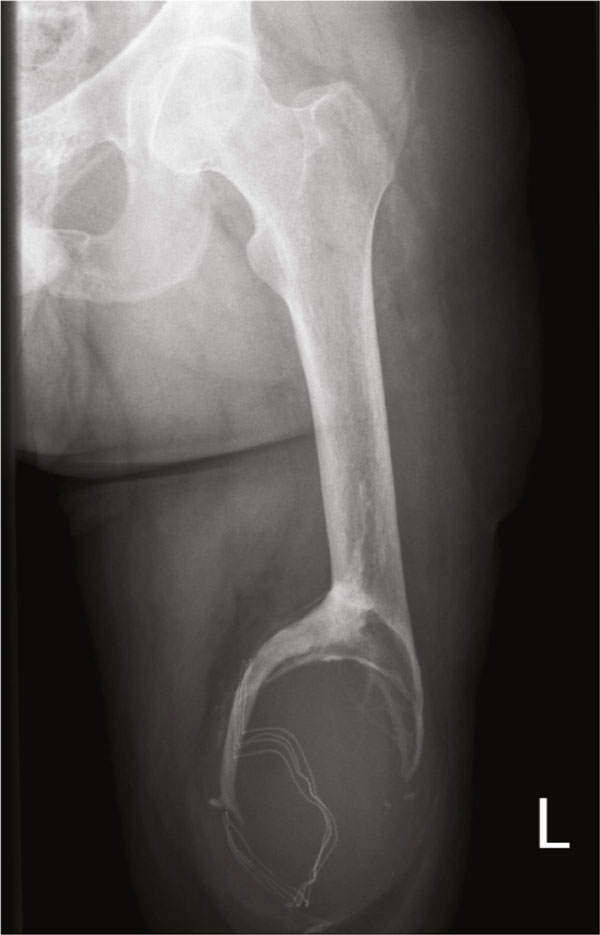
**Roentgenogram upper leg with gauze in situ**.

A 29-year-old Caucasian man was seen in our department for a second opinion because of asymmetrical walking pattern and progressive sourness in his amputated leg. He was referred from a rehabilitation center where a standard roentgenogram showed evidence of a gauze in situ near the distal femur stump with progressive ossifications (Figure 1).

His medical history noted a severe high energy trauma seven years ago. He had sustained multiple injuries among which a severe crush injury of the left leg with heavy bleeding for which an acute amputation was performed.

After six weeks he could be discharged from the hospital and was measured an advanced prosthetic leg for optimal rehabilitation. It took him 3 months to walk without aids. Two years later he fell on his stump in the bathroom. An X-ray was made of the left upper leg stump to rule out any fracture. The radiologist reported no fracture, but a swelling of soft tissue with calcifications at the femoral cutting edge. The report also mentioned that at the time of roentgenography, the stump was covered with a gauze, because a radio-opaque marker was visible on the X-ray. In the conclusion of the X-ray report, no mention was made on the gauze fibroma.

His painful stump recovered in a few weeks but five years later, he consulted his rehabilitation physician for asymmetrical walking pattern with soreness of the stump. A standard roentgenogram revealed progressive calcifications compared to the previous study. The radio-opaque marker was recognized as a retained surgical gauze which was left behind during initial surgery. The progressive ossifications were thought to cause his complaints. He was directed to our department for re-evaluation. Clinical examination showed an otherwise healthy man with a prosthetic leg with vacuum fitting. His walking pattern was slightly asymmetrical due to partial weight-bearing of his artificial leg. His left leg stump showed obvious thickening at the distal end. No painful or sharp masses could be palpated and no skin defects were noted, let alone multiple scars from previous trauma and surgery.

To exclude malignant tumor growth, magnetic resonance imaging showed a benign granuloma of 12.6 × 10.0 centimeters with remodelling of the ossal part of the femur around the tumor. This was interpreted as a physiological reaction of the leg to encapsulate the foreign body. The suspected surgical sponge was not clearly identified from the surrounding granuloma.

Most effective treatment options would be to completely remove the calcifications including granuloma with surgical sponge. The patient however had experienced much advantage from the firm swelling of his stump and did consent. His prosthetic leg fitted well in time because of these progressive ossifications around the gauze. Therefore it was decided to only remove the gauze, but not the ossifications nor the granuloma, to remove the trigger for further progression of the ossifications, but to maintain the form of the stump.

**Figure 2:**
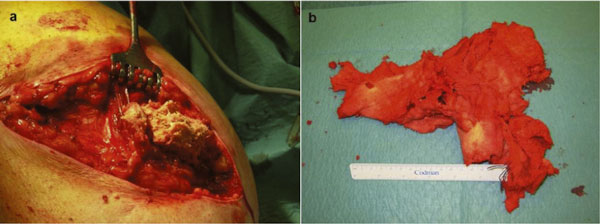
**(a) Gauze removed from fibroma**. **(b) **Aspect and dimensions of gauze.

**Figure 3:**
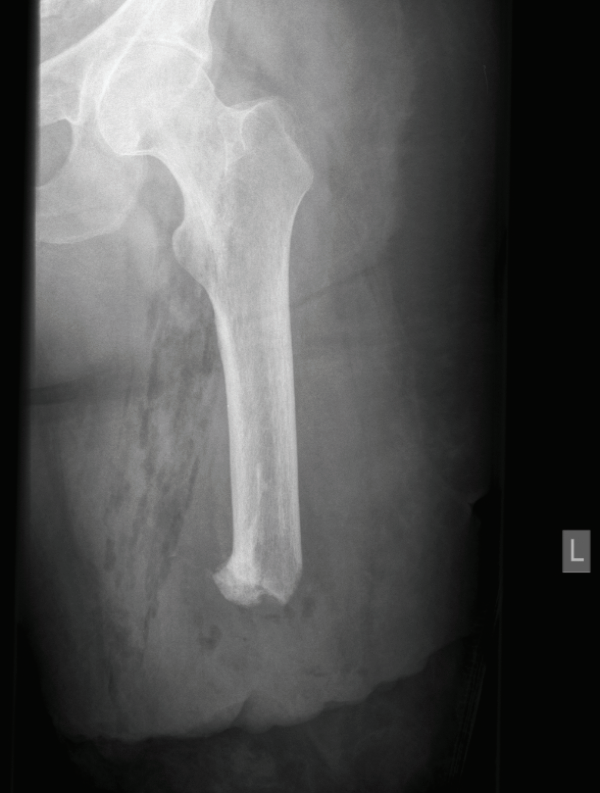
**Roentgenogram upper leg after removal of calcifications**.

At surgery, the distal end of the stump was opened through an old scar. A well organized, firm, brownish tumor was opened which appeared to have a wall of around two centimetres. Brownish, serous fluid was drained and sent for culture of micro-organisms. Remnants of a partly disintegrated surgical sponge of 40 × 40 centimeters were removed with curettage of the granuloma (Figure 2a and Figure 2b). The wound was closed in layers with vacuum drainage. At the fifth postoperative day the patient developed fever and a swollen painful stump for which incision and drainage of an infected seroma with *staphylococcus aureus *was necessary. The postoperative course was further complicated by infectious complications and shortening of the femoral end with removal of all calcifications could not be avoided (Figure 3). Disturbance of wound healing and treatment with vacuum assisted closure devices characterized the clinical course. Five months after initial removal of the gauze, a definitive stump correction was carried out without complications and a rehabilitation program with a new prosthesis could be initiated.

## Discussion

In literature only few cases are described of retained gauze in a limb, years after initial surgery [1,4-7]. If searched in Pubmed by 'gossypiboma' 109 hits are found. When these hits are analysed, 127 cases of gossypiboma can be listed and classified to localisation. Most cases are described in abdomen (58.3%) and thorax (15.7%), but none in an amputated leg (Table 1).

**Table 1:** Localizations of retained gauzes in literature

Localisation	Number	%
Abdomen	74	58.3
Thorax	20	15.7
Pelvic	10	7.9
Spine	5	3.9
Leg	4	3.1
Hip	4	3.1
Retroperitoneal	3	2.4
Breast	2	1.6
Ureter	1	0.8
Neck	1	0.8
Shoulder	1	0.8
Peritoneum	1	0.8
Intracranial	1	0.8
**Total**	**127**	**100**

Radiological differentiation between a gossypiboma and a malignant musculoskeletal tumour is difficult, because they might present with variable spectrum of skeletal changes as osteolytic, periostal and osteoblastic reactions. Retained sponge may be overlooked, unnecessarily invasive investigations might take place, or an erroneous diagnosis may be made [1,3,5,6]. Malignancy in our case was not suspected because of swelling in the soft tissue on the radiography. This was confirmed by MRI. In some cases destruction, osteolysis and calcification were observed on radiographs [1,5]. In our case, calcification and osteolysis, caused by a surgical sponge with a radio-opaque marker was found at the distal femur on radiographs.

It is obvious that in this case the retained gauze was apparently found on radiography two years after amputation when he fell on his stump in the bathroom and a radiogram was performed to rule out a fracture, but it was not interpreted as a retained surgical sponge and thus no treatment plan was deployed. Apparently the clinician who referred him to the radiologist did not think of a gossypiboma. Also, the important information on the fibroma in the stump caused by the gauze was not mentioned in the conclusion. The patient was referred to another hospital for advice, where no treatment plan was made. Insufficient communication between physicians did not lead to adequate treatment. Unfortunately, other case reports do not mention whether radiological investigation preceded discovery of the retained gauze at surgery or whether there was miscommunication between treating physicians. Underreporting of gossypibomas and complications in general is common in medical literature [8,9].

## Conclusion

A retained surgical sponge after amputation may lead to fibroma. In this case the gauze lead to mild dysfunction of the prosthetic leg with asymmetrical walking pattern due to pain. More importantly, when abnormal findings on previous radiographs would have been interpreted correctly, the gauze could have been removed much earlier, preventing the massive ossifications to occur. This fact again points out the importance to think of gossypiboma whenever radio-opaque markers, fibromas or unexpected ossifications occur on routine radiographs.

## Consent

Written informed consent was obtained from the patient for publication of this case report and accompanying images. A copy of the written consent is available for review by the Editor-in-Chief of this journal.

## Competing interests

The authors declare that they have no competing interests.

## Authors' contributions

IK was the treating physician while patient was admitted in our department and drafted the manuscript. JF was involved in design of the case report, he conceived the original idea of the case report, conducted the operation detailed and has been involved in critically revising the manuscript.
